# Numb-like (NumbL) downregulation increases tumorigenicity, cancer stem cell-like properties and resistance to chemotherapy

**DOI:** 10.18632/oncotarget.11553

**Published:** 2016-08-23

**Authors:** José M. García-Heredia, Eva M. Verdugo Sivianes, Antonio Lucena-Cacace, Sonia Molina-Pinelo, Amancio Carnero

**Affiliations:** ^1^ Instituto de Biomedicina de Sevilla (IBIS), Hospital Universitario Virgen del Rocio, Universidad de Sevilla, Consejo Superior de Investigaciones Cientificas, Seville, Spain; ^2^ Department of Vegetal Biochemistry and Molecular Biology, University of Seville, Seville, Spain; ^3^ Present address: Instituto de Investigación Hospital 12 de Octubre, Madrid, Spain

**Keywords:** NumbL, Notch, cancer stem cells, tumor suppressor, tumorigenicity

## Abstract

NumbL, or Numb-like, is a close homologue of Numb, and is part of an evolutionary conserved protein family implicated in some important cellular processes. Numb is a protein involved in cell development, in cell adhesion and migration, in asymmetric cell division, and in targeting proteins for endocytosis and ubiquitination. NumbL exhibits some overlapping functions with Numb, but its role in tumorigenesis is not fully known. Here we showed that the downregulation of NumbL alone is sufficient to increase NICD nuclear translocation and induce Notch pathway activation. Furthermore, NumbL downregulation increases epithelial-mesenchymal transition (EMT) and cancer stem cell (CSC)-related gene transcripts and CSC-like phenotypes, including an increase in the CSC-like pool. These data suggest that NumbL can act independently as a tumor suppressor gene. Furthermore, an absence of NumbL induces chemoresistance in tumor cells. An analysis of human tumors indicates that NumbL is downregulated in a variable percentage of human tumors, with lower levels of this gene correlated with worse prognosis in colon, breast and lung tumors. Therefore, NumbL can act as an independent tumor suppressor inhibiting the Notch pathway and regulating the cancer stem cell pool.

## INTRODUCTION

NumbL, or Numb-like, is a close homologue of Numb and is part of an evolutionary conserved protein family implicated in some important cellular processes, including cell adhesion and migration, asymmetric cell division, and targeting proteins for endocytosis and ubiquitination [1–7]. Numb was originally identified as a membrane-associated protein (dNumb) in *Drosophila* mutants with severe defects in neuron development [8]. This protein is asymmetrically segregated during telophase to only one daughter cell, acting as a cell fate determinant. The daughter cell with dNumb will differentiate while the cell without dNumb will remain in an undifferentiated state [6]. dNumb is evolutionarily conserved, with its mammalian homologues encoded by two genes, Numb and NumbL [9]. Although vertebrate Numb also shows asymmetric distribution in cells, NumbL has been shown to be symmetrically distributed in cytoplasm, which differentiates it from its close homologue Numb [10]. In addition, Numb is ubiquitously expressed during development, while NumbL is more restricted to the developing central nervous system [7, 10–12]. These and other studies suggest that Numb and NumbL, despite their similarities, maintain at least some independent functions. This is more obvious during development in mice. NumbL removal does not have any apparent effects in mice lacking the gene, with the exception of a reduction in female fertility [7]. However, Numb knock-out or double Numb/NumbL knockouts lead to demise in mice during development; the phenotype with double knock-out is more severe in terms of early embryonic lethality, showing that Numb/NumbL partially overlap but have some important differences [7, 13, 14].

Accumulating evidence suggests a potential role of Numb as a tumor suppressor [15, 16], including inhibition of the Notch signaling pathway [17] and the stabilization of p53 [18, 19]. Loss of Numb was associated with poor prognosis in malignant pleural mesothelioma [20]. Overexpression of Numb significantly inhibits proliferation, enhances apoptosis and increases sensitivity to cisplatin [20]. In Numb-negative breast and clear cell renal carcinoma cells, ectopic overexpression of Numb suppresses proliferation [21, 22]. *In vivo*, RNA interference of Numb in a model of mouse lymphomagenesis accelerates the onset of lymphomas [23]. Loss of Numb expression has also been reported in some types of human cancer, including breast, NSCLC, and salivary gland carcinomas and medulloblastoma [21, 24–26].

However, there is a trend toward higher Numb expression in more malignant tumors in human astrocytomas [27]. Overexpression of Numb has also been observed in cervical squamous carcinoma cells [28], suggesting that Numb may act as an oncogene in certain tissues.

In mammals, six differentially spliced isoforms of Numb, each with different expression patterns, have been described to date, while only one NumbL protein has been identified [29, 30]. This suggests that certain Numb isoforms (isoforms 2, 4, 5 and 6) promote tumor growth [31–33], while the Numb-1 isoform behaves as a tumor suppressor [34] and Numb-3 behaves as an oncogene or tumor suppressor depending on the tissue [35]. This raises the possibility that the role of Numb is isoform-specific.

Considerably less is known about mammalian NumbL and its role in tumorigenesis. NumbL was reported to be downregulated in lung cancer cell lines; its ectopic expression suppresses proliferation and invasion, increasing apoptosis [36]. Similarly, in human glioblastoma cells, the overexpression of NumbL suppressed, while the elimination of NumbL promoted the migration and invasion of glioma cells [37]. However, another report found that increased deregulated expression of NumbL in lung tumor cell lines mediated cell migration and in human tumors correlates with shorter patient survival [38]. Therefore, there are contradictory data about the role of NumbL in cancer progression.

Our aim in this study was to investigate the role of NumbL in tumorigenesis, specifically the control of the CSC phenotype, which has emerged as a preferred target in cancer therapy because of its role in cancer recurrence.

Here we show that NumbL knockdown increases tumorigenic properties in three different cancer cell lines of different origins, cervix HeLa, breast T47D and sarcoma AX, due to Notch pathway activation by stabilizing NICD. NumbL knockdown is sufficient to induce cancer stem cell-like transcription and phenotypic properties, suggesting an important role as a tumor suppressor gene by maintaining the CSC-like phenotype through Notch pathway activation. Furthermore, low levels of NumbL decrease sensitivity to chemotherapy and correlate to a worse prognosis in breast, lung and colorectal tumors.

## RESULTS

### shRNA against NumbL induces protein downregulation and a higher number of colonies in clonogenic assays

**Figure 1:**
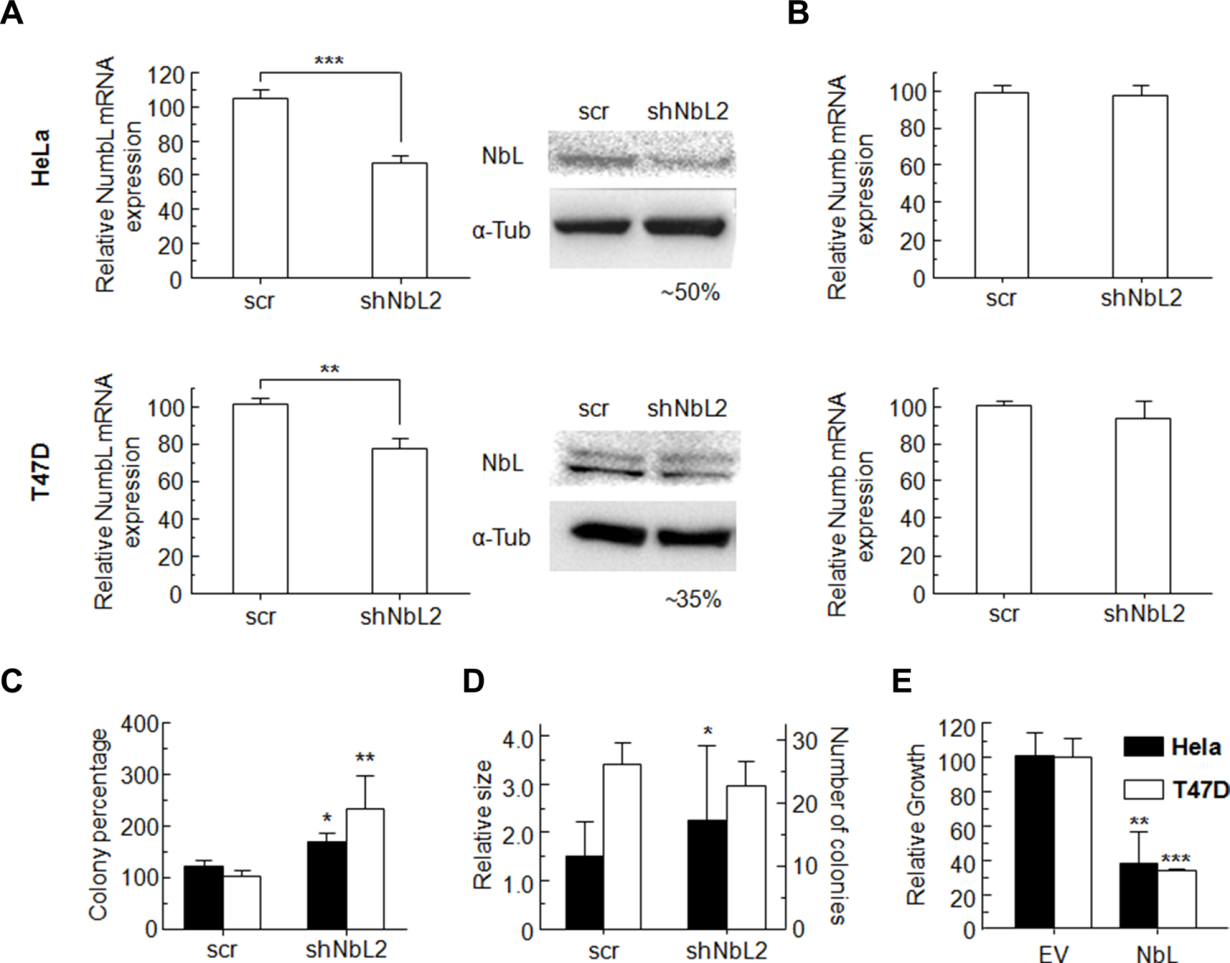
(**A**) Transfection of HeLa or T47D cells with NumbL-shRNA plasmids induce a decrease in NumbL expression, detected both by RT-qPCR and WB assays. (**B**) This shRNA has no effect on Numb expression as determined by RT-qPCR assays. (**C**) Clonogenicity assays for HeLa and T47D pBABE cells transfected with scrambled or shNbL shRNA2, (**D**) soft agar, (**E**) growth clonability by overexpression of NumbL cDNA. All experiments were repeated a minimum of three independent times in triplicate. All figures include Student's *t*-test for statistical analysis of the data. * = *p* < 0.05; ** = *p* < 0.01; *** = *p* < 0.001.

Human cell lines HeLa and T47D transfected with shRNA against NumbL (shNbL2) showed a decrease in the expression of this protein, both at mRNA and protein levels (Figure 1A). Despite the high degree of similarity between Numb and NumbL, cell lines expressing shNbL2 show no significant changes in Numb expression (Figure 1B).

Clonogenic assays performed at low cell densities showed that NumbL downregulation induces a significant increment in colony numbers, both from HeLa and T47D cells (Figure 1C). Colony numbers from HeLa cells were increased to 140%, while for T47D cells, NumbL downregulation induced a further significant increase up to values close to 230% compared to the control expressing a scrambled shRNA cell line. This increment in colony number has been associated with increased cell survival. Further experiments growing these cells in soft agar also showed a clear increase in the number of colonies growing in cells with downregulated NumbL (Figure 1D). To further confirm the role of NumbL as a tumor suppressor, we overexpressed NumbL cDNA into these cells. The experiment showed a marked reduction in the number of cells able to form colonies (Figure 1E). These data confirm the potential of NumbL to act independently as a tumor suppressor.

### NumbL downregulation activates the Notch pathway

**Figure 2:**
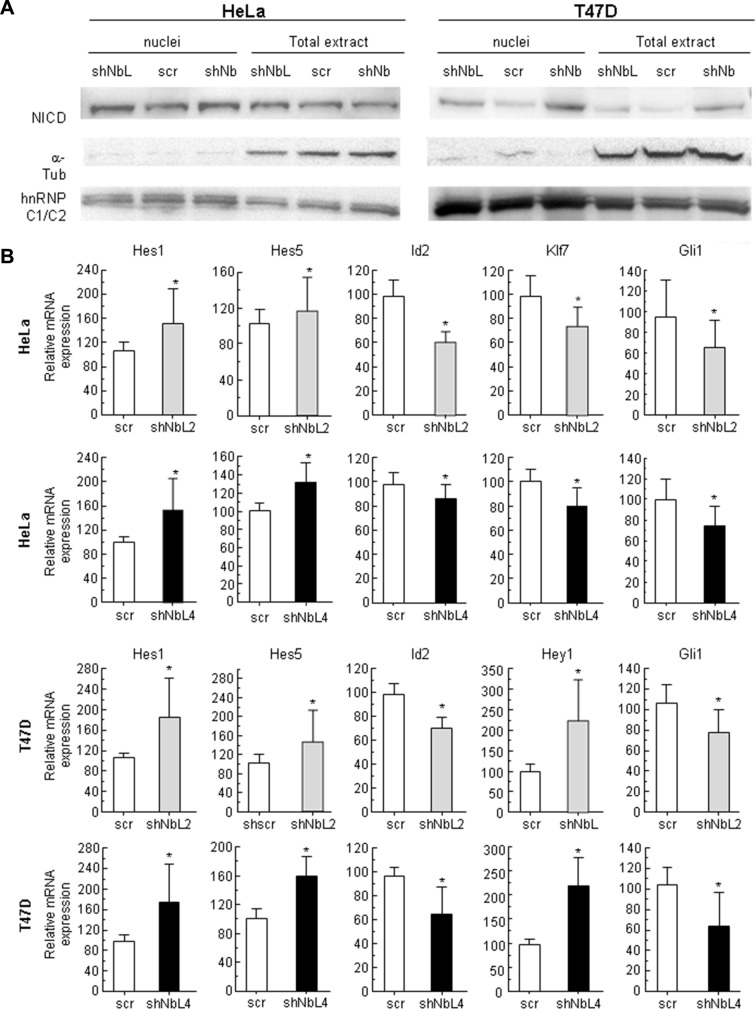
NumbL downregulation activates Notch signaling (**A**) NICD localization in nuclei and total extracts in HeLa and T47D cells. (**B**) Expression of Notch pathway-related genes. Two different shRNAs (shNbL2 and shNbL4) were used to eliminate possible off-target effects. The sequence of both shRNAs is shown in M&M. All experiments were repeated a minimum of three independent times in triplicate. All figures include Student's *T* test for statistical analysis of the data. * = *p* < 0.05; ** = *p* < 0.01; *** = *p* < 0.001.

Due to its close relationship to Numb and the known connection between Numb and Notch, we decided to analyze the effect of NumbL downregulation on the Notch pathway. We detected an increment in nuclear NICD in HeLa and T47D cells with low levels of NumbL compared to the control cells (Figure 2A). This increase is similar to the increase observed upon Numb downregulation by shRNA (Figure 2A). As a consequence of NICD nuclear translocation, the Notch pathway is activated. We found Hes1/Hes5 activation and Gli1 inhibition, as well as Klf7 and Id2 inhibition (Figure 2B), all well-known downstream genes regulated by NICD and Notch pathways [39–42]. Taken together, these data suggest that NumbL downregulation induces Notch pathway activation by protecting NICD from degradation.

### Acquisition of stem cell-like properties due to NumbL downregulation

**Figure 3:**
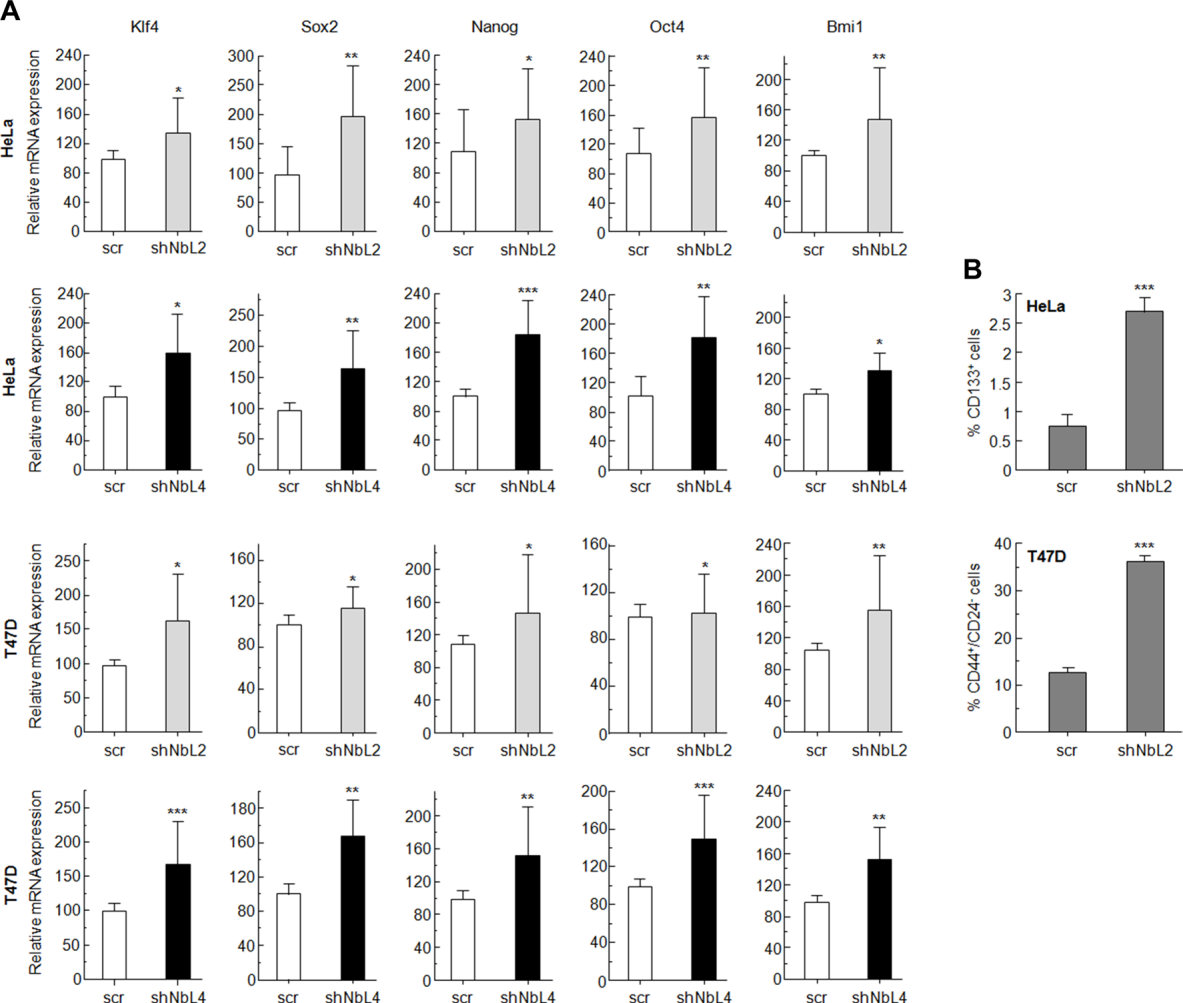
NumbL downregulation activates Notch stem-cell like properties in tumor cells (**A**) Quantitative PCR results with genes related to stem-cell like properties. Two different shRNAs (shNbL2 and shNbL4) were used to eliminate possible off-target effects. Sequence of both shRNAs is shown in M&M. (**B**) CD44^+^/CD24^−^ and CD133^+^ FACS of T47D and HeLa cells, respectively. All experiments were repeated a minimum of three independent times in triplicate. All figures include Student's *T* test for statistical analysis of the data. * = *p* < 0.05; ** = *p* < 0.01; *** = *p* < 0.001.

It has been previously established that activation of the Notch pathway induces EMT and cancer stem cell-like properties [43]. Using RT-qPCR, we analyzed the expression of some genes that have been connected with the acquisition of EMT and stem cell-like properties. NumbL downregulation induced a clear increment in EMT-dependent transcript Snai1 and Twist1 in Hela and T47D cells (Supplementary Figure S1A). In both HeLa and T47D cells, we also observed a significant induction of Klf4, Sox2, Nanog, Oct4 and Bmi1 genes (Figure 3A). These genes have been previously related to stem cell properties [44–46]. The higher expression of these genes showed that downregulation of NumbL and, consequently, Notch pathway activation, turn cells into a certain de-differentiated state typical of stem-like cells.

In addition, FACS analysis of HeLa and T47D cells with downregulated NumbL showed a higher expression of phenotypic markers associated with stem cells (Figure 3B and Supplementary Figure S2). For T47D cells, a breast tumor cell line, we used double CD44^+^/CD24^−^ staining, which has been extensively used to identify cancer stem cells [47]. In this case, NumbL downregulation increased the percentage of CD44^+^/CD24^−^ from 13.6% of scrambled cells to 36.6% of cells expressing NumbL shRNA (Figure 3B). In addition, CD44 has been characterized as a target gene activated by the Notch signaling pathway [48]. In the case of HeLa cells, we used the CD133 stem cell marker to differentiate the CSC subpopulation [49]. CD133^+^ HeLa cells increased from 0.8% in control cells to 2.7% in cells expressing NumbL shRNA, indicating that NumbL downregulation in HeLa cells also induces the phenotypic markers of CSC-like cells.

### Analysis of cancer stem cell-like properties of cells with downregulated NumbL

**Figure 4:**
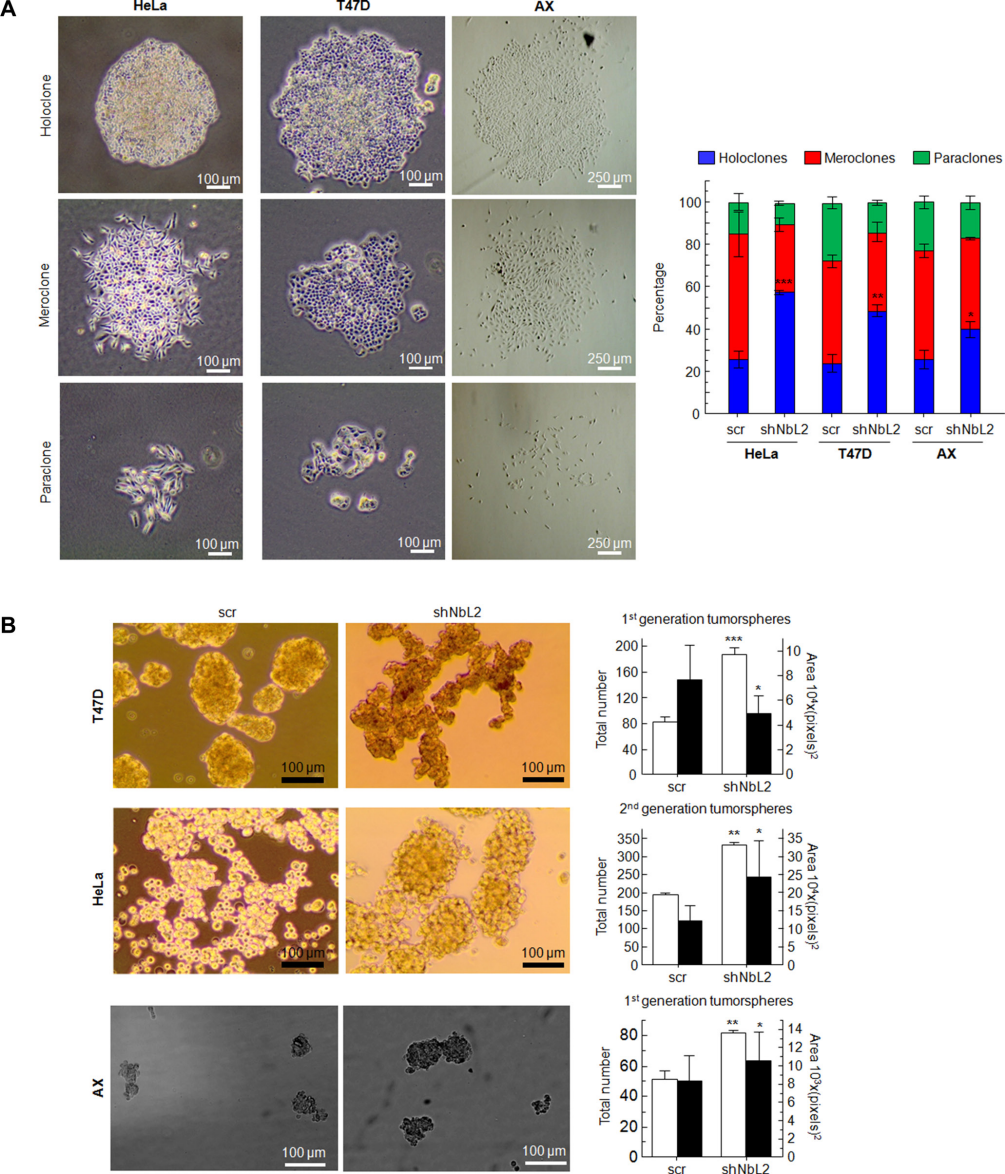
(**A**) Type and percentage of each of the different clones observed for HeLa, T47D and AX cells. Figure shows typical colonies (left) and the percentage of each type in all cases (right). (**B**) Tumorsphere assay for HeLa cells (2nd generation), T47D cells (1st generation), and AX low passaged sarcoma cells (1st generation) showing typical colonies (left) and both total number and size of tumorspheres (right). All experiments were repeated a minimum of three independent times in triplicate. All figures include Student's *T* test for statistical analysis of the data. * = *p* < 0.05; ** = *p* < 0.01; *** = *p* < 0.001.

To this end, we cultured cells at low density to form independent colonies from individual clones. These clones have been previously classified as holoclones, meroclones and paraclones based on their ability to reconstitute a tumor from a single cell [50, 51]. Basically, holoclones are considered to be derived from stem cells, while paraclones are differentiated cells that are incapable of reconstituting a culture (Figure 4A). Meroclones are intermediate phenotypes between holo- and paraclones. The percentage of holoclones in shNbL cells was increased from 30% to 60% in HeLa cells, while in T47D this increment was from 30% to 50% (Figure 4A).

Next, we tested the ability of cells with reduced levels of NumbL to form tumorspheres, another surrogated assay for the cancer stem-like phenotype. The cells were seeded and visualized five days after seeding. T47D tumor cells formed spheres at this stage, considered as 1st generation tumorspheres. There were morphological differences between tumorspheres from scrambled or shNbL cells carrying NumbL shRNA2 (Figure 4B). The number of tumorspheres derived from cells with reduced NumbL was significantly higher compared to the control scrambled shRNA. The analysis of the 2nd generation tumorspheres derived from HeLa cells showed even more evident differences between control and NumbL downregulated cells (Figure 4B). At this stage, only shNbL2 cells showed real tumorspheres (Figure 4B). In this case, the spheres were also larger when NumbL was downregulated.

Finally, we decided to downregulate NumbL in a low passaged sarcoma cell line, AX. This cell line is close to the primary sarcoma tumor (liposarcoma) because it has been passaged less than 20 times from the original explant. This cell line behaves in a similar fashion to Hela and T47D regarding the increase of the stem cell-like pool upon NumbL downregulation (Figure 4).

### Overexpression of NumbL cDNA reduced Notch and stem cell gene transcription and stem cell-like properties

**Figure 5:**
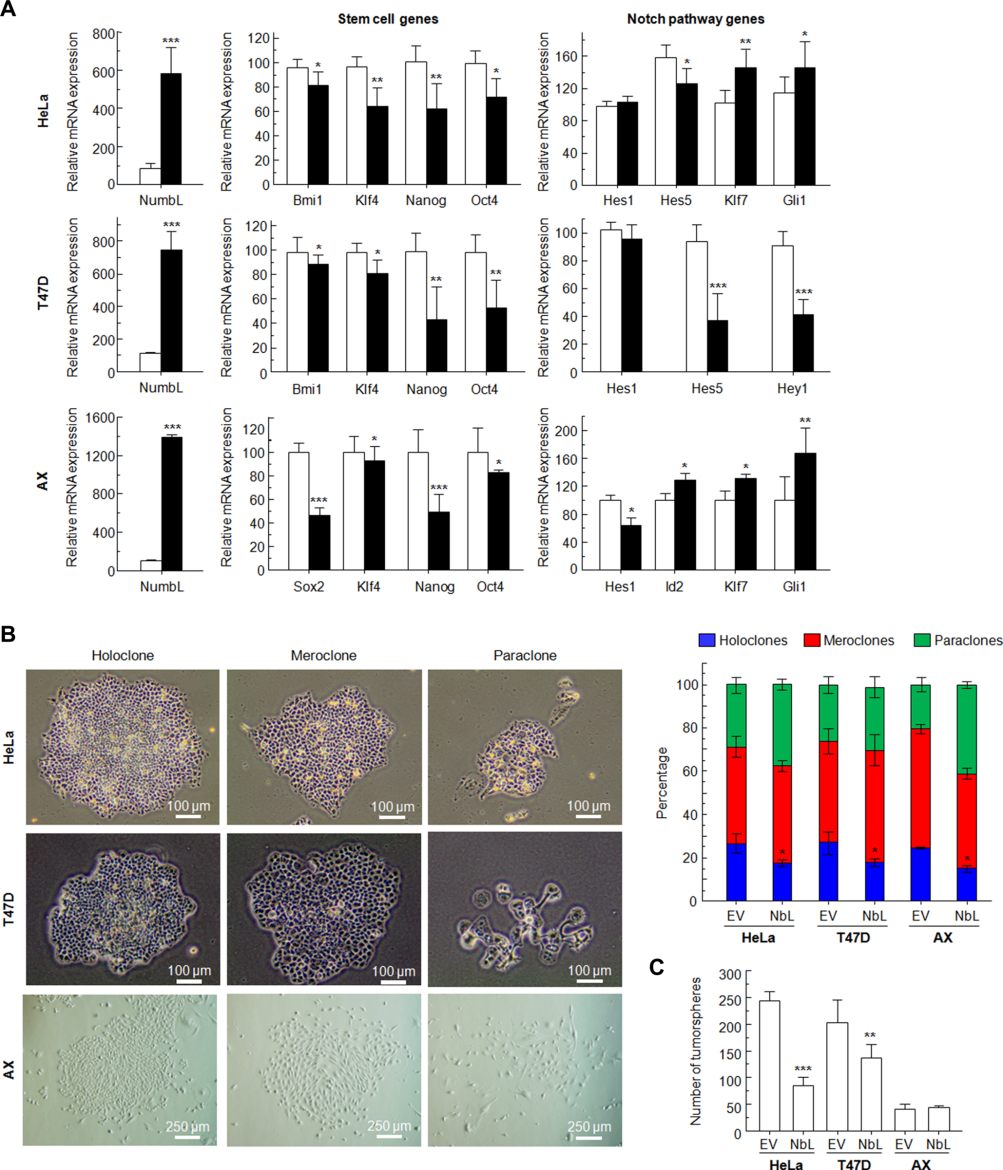
Overexpression of NumbL cDNA in HeLa, T47D and AX cells (**A**) Overexpression of NumbL cDNA downregulates stem- and Notch-pathway related genes. (**B**) Type and percentage of each of the different clones observed for HeLa, T47D and AX cells upon overexpression of NumbL cDNA. Figure shows typical colonies (left) and the percentage of each type in all cases (right). (**C**) Tumorsphere assay for HeLa cells (2nd generation), T47D cells (1st generation), and AX low passaged sarcoma cells (1st generation) showing total number of tumorspheres. All experiments were repeated a minimum of three independent times in triplicate. All figures include Student's *T* test for statistical analysis of the data. * = *p* < 0.05; ** = *p* < 0.01; *** = *p* < 0.001.

To fully confirm the role of NumbL as a tumor suppressor acting on the Notch pathway and stem cell gene transcription, we decided to overexpress NumbL cDNA into these three cell lines and assess transcription and behavior. The overexpression of NumbL triggers a decrease in the stem cell genes and the inactivation of the Notch pathway (Figure 5A). As a consequence, NumbL overexpression decreases the number of holoclones (Figure 5B) and tumorspheres (Figure 5C), finally leading to a decrease in colony growth (Figure 1E).

**Figure 6:**
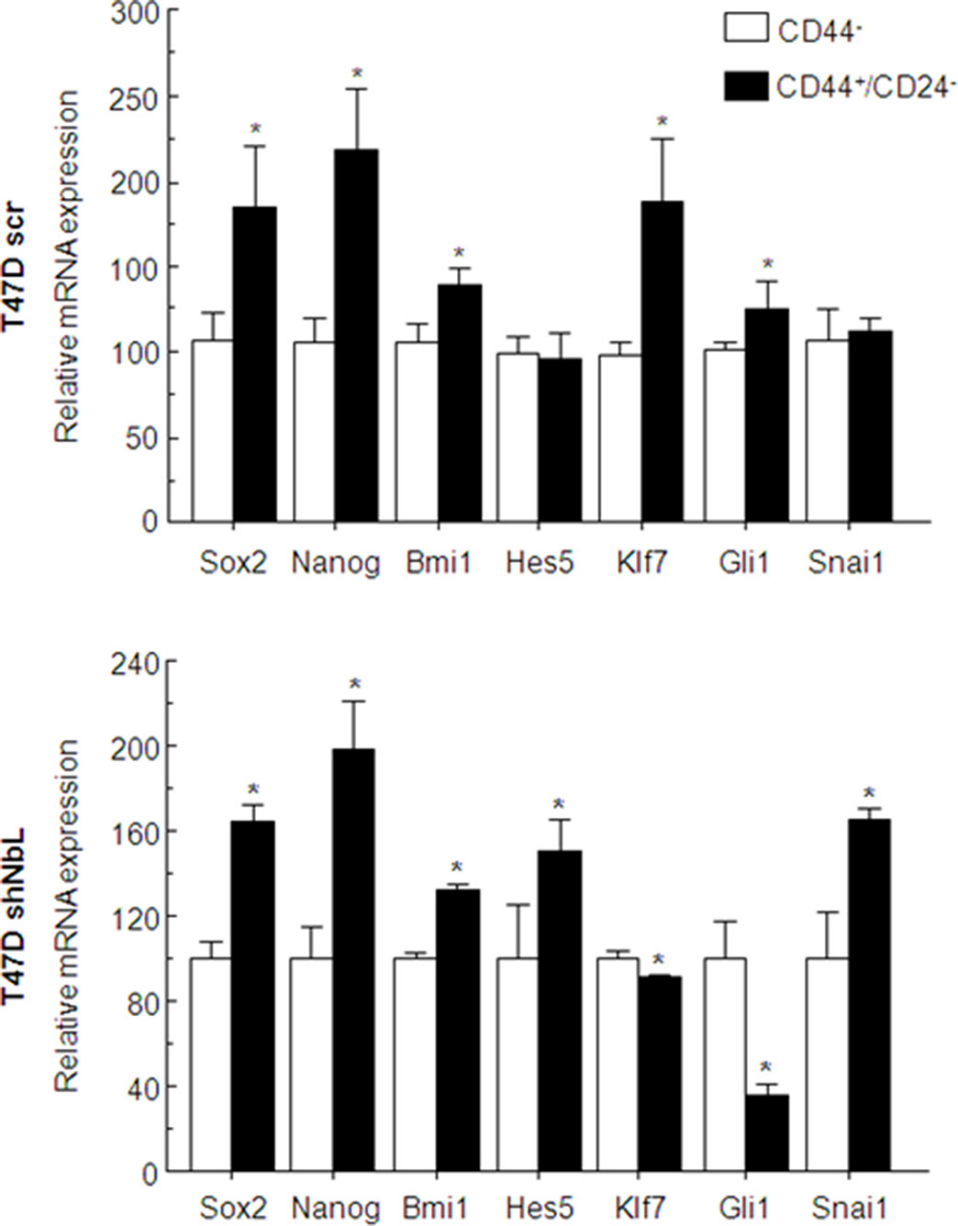
T47D control cells or T47D cells expressing the shNbl2 were selected for CD44^+^/CD24^−^ (solid black bars) or CD44^−^ (open bars) subpopulations Transcriptional analysis of stem- or Notch-related genes was performed by qRT-PCR. All experiments were repeated a minimum of three independent times in triplicate. All figures include Student's *T* test for statistical analysis of the data. * = *p* < 0.05; ** = *p* < 0.01; *** = *p* < 0.001.

Finally, to explore the role of Notch activation, we separated the stem cell-like subpopulation from T47D, the CD44^+^/CD24^−^ cells from the CD44^−^ cells, and analyzed whether there was differential activation of the Notch pathway in these cells regarding the levels of NumbL. In the T47D control cells, we observed that stem cell gene transcription was increased in CD44^+^/CD24^−^ cells; however, Notch-dependent transcription was inhibited, as denoted by the normal levels of Hes5 and the activation of Klf7 and Gli1 (Figure 6, upper panel). In the T47D with downregulated levels of NumbL, we found a marked activation of the Notch pathway in CD44^+^/CD24^−^ cells, as denoted by the activation of Hes5 and the repression of Klf7 and Gli1 (Figure 6, bottom panel). We also observed differences in the levels of Snai1, suggesting that the EMT was dependent on Notch pathway activation.

### NumbL downregulation increases chemoresistance

**Figure 7:**
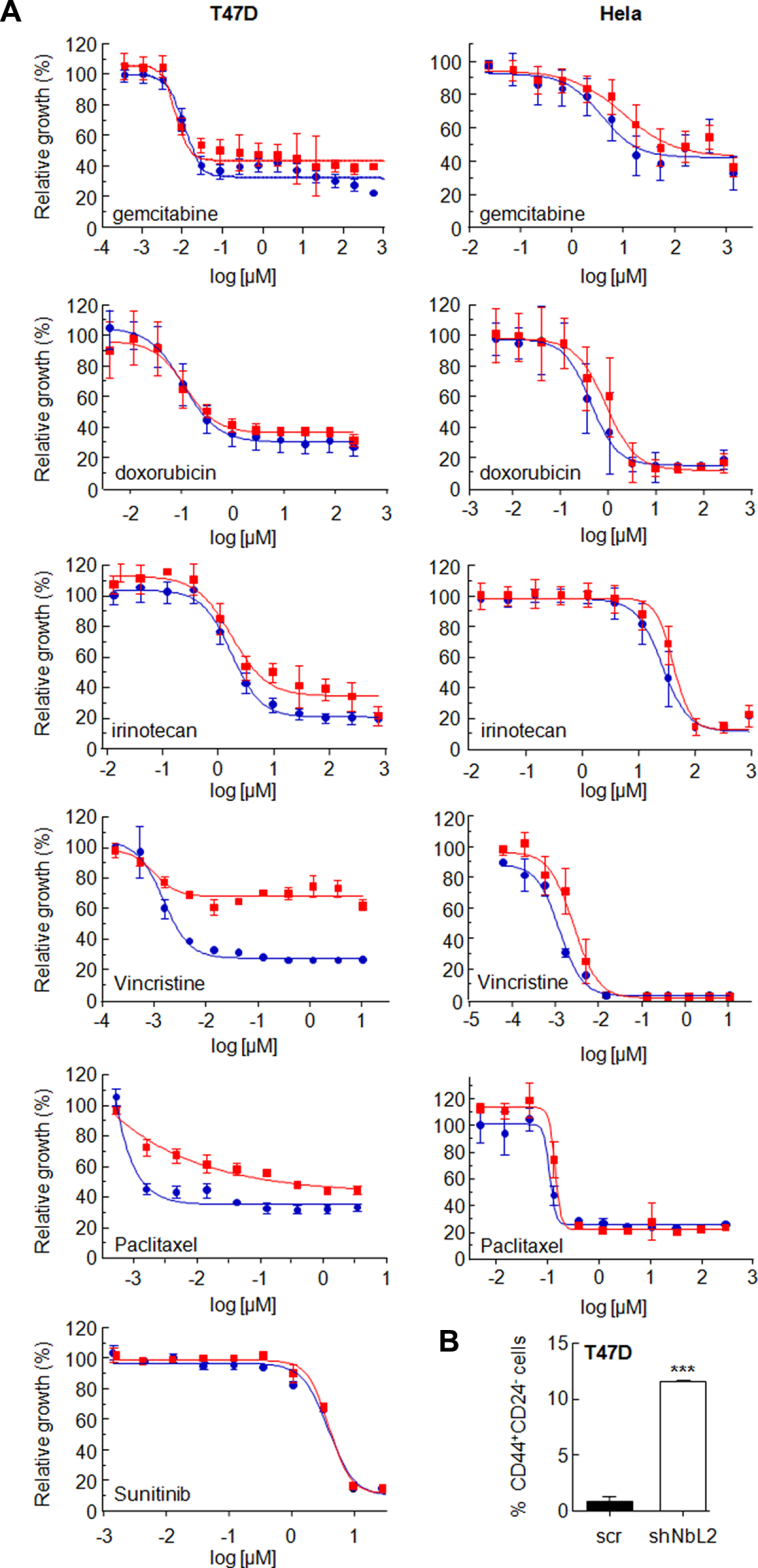
(**A**) NumbL downregulation induces resistance to different chemotherapeutic drugs. Hela and T47D cells carrying scrambled (blue circles) or NumbL shRNA2 (red squares) were treated with 11 different doses of the indicated drug for 96 hrs. A minimum of three independent experiments in triplicate were performed to obtain the average values (Table 1). The figure shows one representative experiment. Bars indicate SD of the triplicate samples. (**B**) T47D cells were treated with vincristine and were subjected to flow cytometry analysis to detect the population of CD44^+^/CD24^−^ cells. The percentage of CD44^+^/CD24^−^ cells is shown. All experiments were repeated a minimum of three independent times in triplicate. All figures include Student's *T* test for statistical analysis of the data. * = *p* < 0.05; ** = *p* < 0.01; *** = *p* < 0.001.

It has been proposed that the extent of chemoresistance is related to the percentage of CSCs in the tumor. Since downregulation of NumbL increases the CSC-like phenotype in culture, we tested whether this phenotype is also associated to chemoresistance. To this end, we treated Hela and T47D cells carrying NumbL shRNA (shNbL2 cells) or scramble (scr) to different doses of several chemotherapeutic drugs used in clinical regimens: doxorubicin, irinotecan, 5-FU and gemcitabine. After IC50 calculations, we observed that Hela cells with NumbL downregulated showed on average a two-fold increase in IC50 (Table 1), confirming the increase of resistance of these cells to the drug tested. In T47D, we did not observe an increase in IC50 throughout the different experiments performed (Table 1); however, we detected in most cases an increase in the percentage of cells resistant to the treatment (Figure 7). To explore this in more depth, we subjected T47D to new drugs most commonly used in breast tumors, including vincristine, capecitabine and paclitaxel. In all cases NumbL-downregulated T47D were more resistant to treatment (Table 1). This is especially relevant in the case of vincristine and paclitaxel, in which a large percentage of cells remained resistant to the treatment (Figure 7A). This resistance is not a general characteristic of the newly generated cell line with low NumbL because the sensitivity remained identical to control cells with other treatments such as sunitinib (Figure 7A). Because mammary tumor cells showed the highest ratio of resistance to vincristine treatment, we treated T47D cells with vincristine and subjected them to flow cytometry analysis to detect the population of CD44^+^/CD24^−^ cells. The results indicated that resistant cells have an increased proportion of CD44^+^/CD24^−^ cells being enriched in cancer stem-like cells, mainly when NumbL is downregulated (Figure 7B).

**Table 1:** IC50 (μM) values for Hela and T47D expressing shRNA scramble (scr) or shRNA against NumbL (shNbl) of different drugs commonly used in cancer chemotherapy

HeLa			T47D	
	scr	shNbL	scr	shNbL
**Doxorubicin**	0.4 ± 1.12	0.8 ± 1.24	0.11 ± 1.09	0.12 ± 1.21
**Irinotecan**	27.6 ± 1.06	43.65 ± 1.09	2.21 ± 1.1	2.32 ± 1.34
**5-FU**	46 ± 1.11	94.4 ± 1.12	2.46 ± 2.31	3.54 ± 2.18
**Gemcitabine**	3.84 ± 1.46	10.23 ± 1.58	0.0091 ± 1.24	0.0067 ± 1.1
**Vincristine**	1.26 ± 0.35	2.71 ± 0.56	0.001 ± 0.001	Not reached
**Sunitinib**	NT	NT	2.46 ± 2.31	3.54 ± 2.18
**Capecitabine**	NT	NT	466,96 ± 45.26	683,26 ± 70,36
**Paclitaxel**	0.125 ± 0.05	0.142 ± 0.03	0.0169 ± 0.001	0.9 ± 0.1

### NumbL downregulation in human tumors

Our previous data strongly suggest that NumbL may behave as a tumor suppressor in human tissue. To explore this possibility, we searched public array databases (through Oncomine) for the possible downregulation of this gene expression in tumors. We found transcriptomic analysis showing that when comparing normal colon tissue to colorectal tumors, the tumors showed consistently lower expression of this gene (Supplementary Figure S3). Breast and lung tumor databases also exhibited a variable number of tumor samples with downregulated NumbL mRNA levels, which does not account for a significant decrease of the total tumor population (data not shown). This decrease in NumbL transcription may be a consequence of promoter methylation. To explore this point, we analyzed our own cohort of lung tumors (Supplementary Table S2).

To evaluate the potential role of NumbL promoter methylation in lung cancer, we analyzed the methylation status of the cluster in human lung tissue. The methylation profile of NumbL was evaluated in human tumor samples and compared to non-tumor tissue using the Illumina Infinium Human Methylation 450 BeadChip. The methylation levels in lung cancer versus non-tumor tissue are shown in Table 2. Our data show a significant methylation of the NumbL promoter in lung adenocarcinoma tumors compared to non-tumor lung samples. The same significant NumbL methylation profiles were observed in squamous cell lung cancer. Interestingly, the methylation of the NumbL promoter was more significant in smoker patient samples, while it was non-significant in samples from non-smoker patients. These data reinforce the role of NumbL as a tumor suppressor in human tumors, being downregulated in a variable subset of tumors depending on the tissue.

**Table 2:** Relative levels of methylation in patients with lung cancer relative to the control group

**Adenocarcinoma tumor (mean)**	No tumor control (mean)	Adjusted p value
0,437028882	0,494097649	**3,29371E-05**
**SCC tumor (mean)**	No tumor control (mean)	Adjusted p value
0,425303511	0,511479311	**4,48278E-06**
**Lung tumor Smoker (mean)**	No tumor control (mean)	Adjusted p value
0,431166197	0,50021538	**4,65556E-11**
**Lung tumor non-Smoker (mean)**	No tumor control (mean)	Adjusted p value
0,494918598	0,480170627	0,640782137

Observed methylation changes (log2 ratio). Statistically significant differences (adjusted p-value < 0.05) of methylation levels with respect to the control non-tumor group were considered.

**Figure 8:**
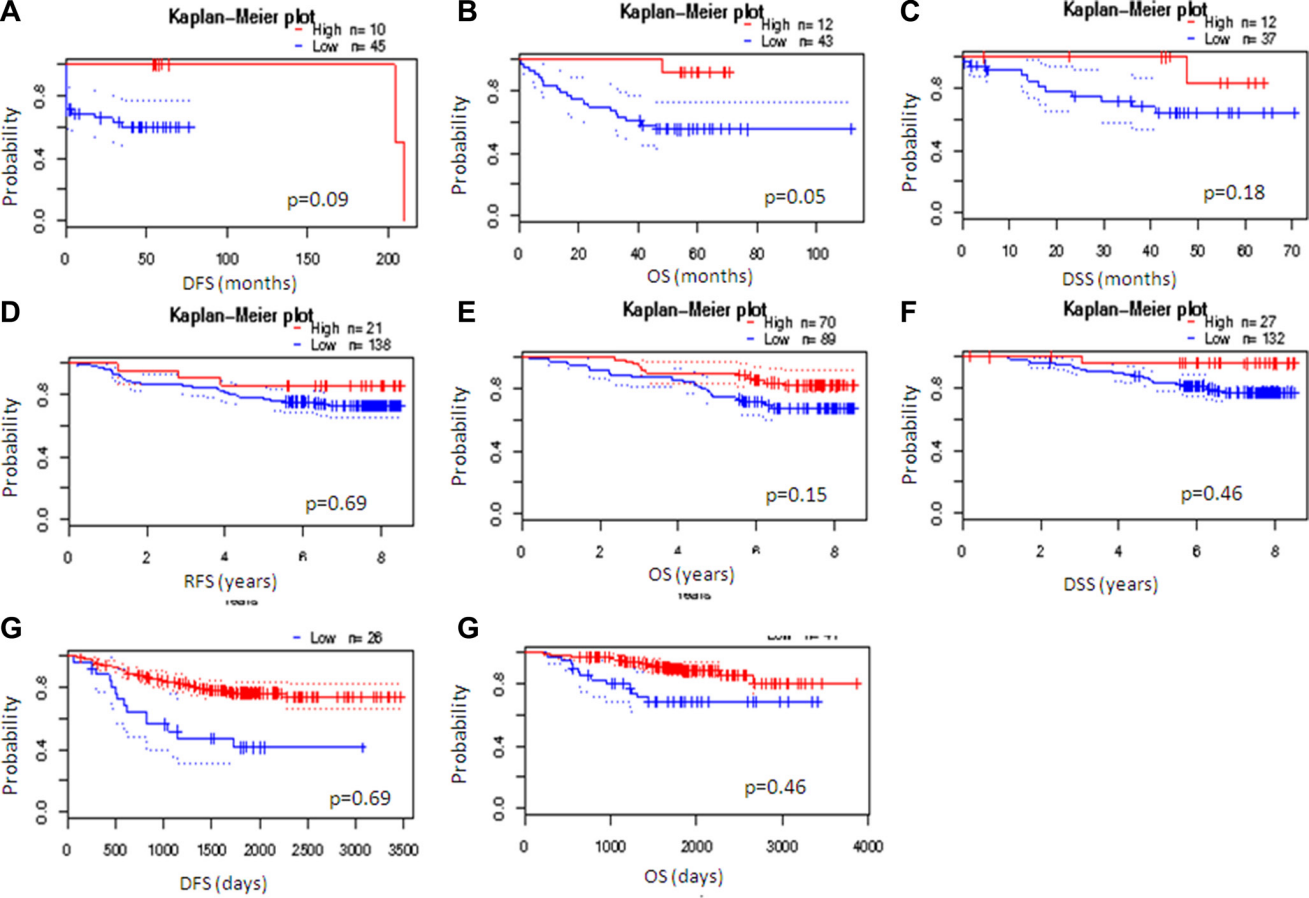
NumbL downregulation results in worse survival in cancer patients Kaplan-Mayer according to NumbL expression in three different tumor types, obtained from three different arrays for each tumor type. Data were obtained from PrognoScan (http://www.abren.net/PrognoScan/). See Supplementary Table S2 for more information.

Furthermore, in colon tumors, low levels of this gene are associated with worse prognosis (Figure 8A–8C and Supplementary Table S2). In breast and lung tumors, the small percentage of tumors with low NumbL mRNA levels also showed worse prognosis (Figure 8D–8H and Supplementary Table S2). However, the overall statistics were not significant values, likely due to the very different number of cases in both arms; yet, a clear tendency can be observed.

**Figure 9:**
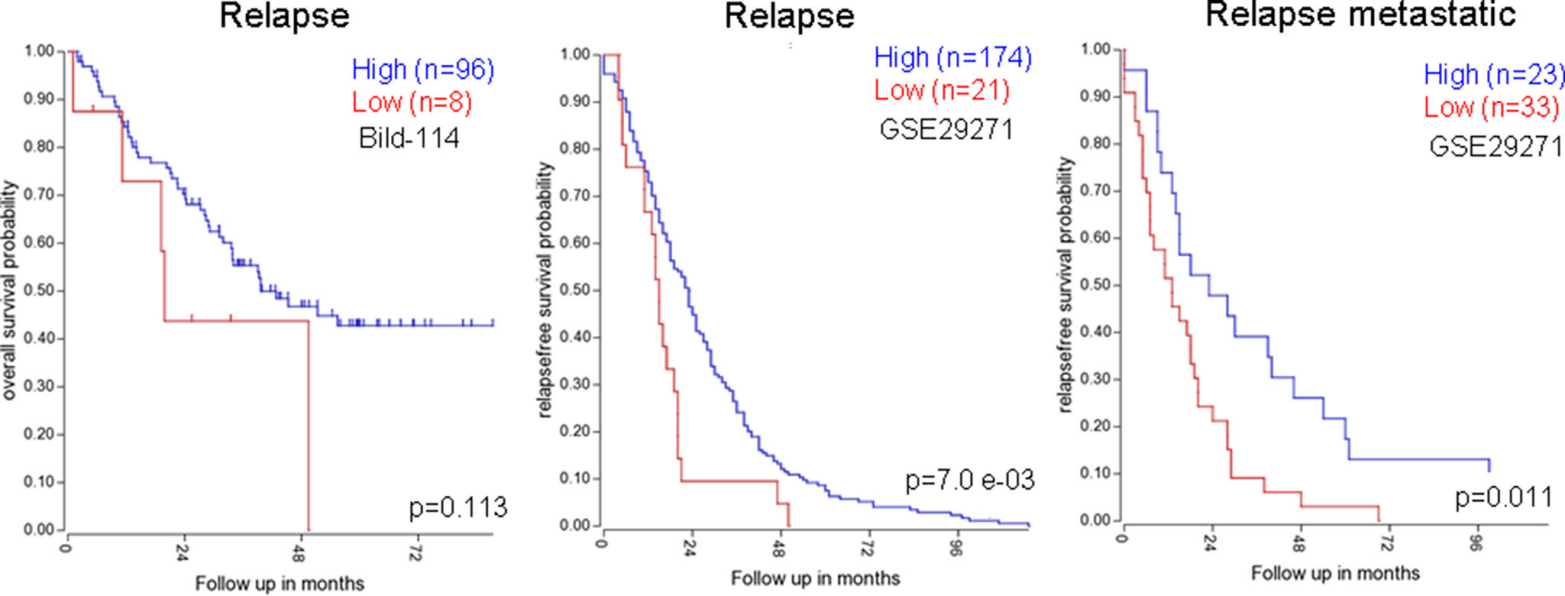
Kaplan-Mayer according to NumbL expression in three different tumor databases, from relapsed only samples Data were obtained from R2: Genomic analysis databases. (http://hgserver1.amc.nl/cgi-bin/r2/main.cgi).

A plausible explanation is that the tumors with downregulated NumbL have a larger number of cancer stem cells that avoid the action of the chemotherapy, thus providing an advantage for tumor relapse. When we specifically selected the relapsed tumors, those with low levels of NumbL had a worse prognosis, at least in breast tumors (Figure 9A and 9B); the relapses were both local and at distant sites (Figure 9C).

## DISCUSSION

NumbL, a closely related homologue to Numb, has been recently linked to cancer. Our results strongly support a tumor suppressor role for NumbL. Downregulating NumbL without changing Numb allows cells to acquire a higher tumorigenic potential due to Notch pathway activation. Therefore, inhibition of only one of the Numb family proteins is sufficient to modify cancer cell properties. Importantly, we found that NumbL downregulation triggers the activation of the Notch pathway, further increasing the EMT and CSC transcriptional markers and CSC-like phenotypes. In human tumors, a number of tumors have a lower NumbL expression than normal tissue. This lower NumbL expression is associated with a poor prognosis and lower patient survival. Our data are consistent with previous work indicating that NumbL downregulation is associated with a higher tumorigenic potential in lung and glioma cancer cell lines [52, 53] and its role as a tumor suppressor.

Furthermore, a partial decrease in NumbL is sufficient to increase Notch pathway activation and the cancer stem-like properties. This suggests that NumbL and its close relative Numb are essential regulators of these properties, acting in a dose-dependent manner. As in the case of Numb, NumbL seems to regulate Notch pathway activity [54, 55]. It is interesting to note that despite the presence of both Numb and NumbL proteins in the cells, the downregulation of one of them is sufficient to launch Notch pathway activation and increase the pool of CSC-like cells. This suggests an important dose effect of combined Numb/NumbL that may have a linear response on the acquisition of the CSC phenotype. This also suggests that downregulation of both proteins may coexist in some very aggressive tumors. This additive effect still needs to be explored, perhaps *in vivo*, to determine whether the accumulative effects are linear or once reaching a certain threshold, there is no further increase in tumorigenicity.

In vertebrates, Numb and NumbL were identified as Notch1 interactor proteins during mouse cortical neurogenesis [10]. Notch and Numb/NumbL proteins have antagonistic roles in cells because while Numb allows cell differentiation, Notch pathway activation mediates the activation of hundreds of genes, some of them associated with stem cell properties [56, 57]. In addition, there is clear evidence that the Notch pathway is activated in cancer cells, allowing them to acquire stem cell-like properties [58, 59]. Numb has been associated with cancer and the acquisition of cancer stem cell properties. dNumb knock-out mutants in Drosophila induce stem cell-like proliferation and tumor development, demonstrating the role of Numb as a tumor suppressor gene [60]. In fact, a hyperactive Notch pathway has been associated with several tumors [61]. However, it has been suggested that Numb may act as an oncogene in human astrocytomas and cervical squamous carcinoma cells [27, 28].

Numb protein promotes Notch1 receptor ubiquitination and NICD degradation through the interaction with the E3 ligase Itch. However, Numb also interacts with members of other RING-type E3 ligases, including LNX, Siah1 and MDM2 [19, 62, 63]. By inducing Numb degradation, these E3 ligases potentiate Notch signaling [62]. It has been demonstrated that enhanced ubiquitination and consequent increased proteosome-mediated degradation account for the loss of Numb in a proportion of breast tumors [21]. Similar mechanisms may exist for NumbL, as its downregulation also triggers nuclear NICD accumulation. Furthermore, we described an increased methylation pattern of a NumbL promoter in lung tumors (Table 2), suggesting another possible mechanism for its downregulation. However, other mechanisms for NumbL inactivation may be relevant for tumorigenesis. For example, the study of cells without both Numb/NumbL exhibited incorrect apical membrane localization for cadherins, causing the loss of adherent junctions [5]. Both proteins can interact through the PTB domain with Lnx2, which acts as a molecular scaffold that may drive Numb/NumbL proteins to a particular subcellular site [64], inactivating its function.

Accumulating evidence suggests a potential role of Numb as a tumor suppressor [15, 16], including inhibition of the Notch signaling pathway [17] and stabilization of p53 [18–23]. On the other hand, Numb and NumbL play different roles in cells, with p53 and sonic hedgehog being differentially affected by Numb and NumbL [65]. NumbL has been described to repress NF-kB induced antitumor activity [52, 53]. Only NumbL interacts through its PTB domain and C-terminal region with Tab2. This NumbL-Tab2 interaction negatively regulates the NF-kB pathway [66]. It has also been shown that NumbL promotes polyubiquitination and degradation of TRAF-6, negatively regulating the NF-kB pathway [67]. Although we found that the increase in this tumorigenic phenotype related to CSCs is related to Notch pathway activation, it is possible that other pathways may also contribute to the activity of NumbL as a tumor suppressor.

The Notch signaling pathway, a critical pathway governing embryonic development, is involved in the maintenance of tumor stem-ness and cancer metastasis. Increased activity of the Notch pathway has been reported in a variety of tumor cell lines and in tumors of different origin, including, lung, colon, breast and prostate tumors, as well as sarcomas, melanomas, leukemias and lymphomas [68–73]. In these studies, Notch activity also appeared to be involved in cancer metastasis by modulating the epithelial-mesenchymal transition (EMT), the tumor angiogenesis processes, and the anoikis resistance of tumor cells [74–76].

Furthermore, we reported that, perhaps as a consequence of the activation of a CSC-like phenotype, low levels of NumbL decrease sensitivity to chemotherapy and are correlated with a worse prognosis in breast, lung and colorectal tumors. We found that decreased levels of NumbL increased resistance to chemotherapy with several drugs, increasing the percentage of stem cell-like cells among the resistant subpopulation. This may explain the low levels of this protein among relapsed tumors, at least in breast cancer.

Our data support the idea of NumbL as a tumor suppressor, sharing this phenotype with its related Numb. In human tumors, loss of Numb expression has also been reported to correlate with worse prognosis in some types of human cancer, including breast, NSCLC, salivary gland carcinomas and medulloblastomas [21, 24–26]. In addition, other tumor suppressor functions of Notch pathway activation have been described [35, 77–79]. It will be interesting to learn whether Numb/NumbL are involved in these apparently contradictory effects on the Notch pathway or are due to NumbL dosage in different tissues or to different functions in other pathways.

In summary, our data help to clarify the role of NumbL in tumorigenesis, suggesting that NumbL acts as a tumor suppressor regulating the Notch pathway, EMT and CSC-like phenotype, and thus contributing to resistance to chemotherapy and worse prognosis.

## MATERIALS AND METHODS

### Cell lines, plasmids and antibodies

T47D and Hela cells were obtained from the European Collection of Authenticated Cell Cultures (ECACC) commercial repository at the beginning of this work. No further authentication was performed in these cell lines. Cells were cultured following the experimental procedure indicated in the ECACC cell line data sheet. Cells were maintained in Dulbecco modified Eagle medium (Sigma) containing 10% fetal bovine serum (Sigma), penicillin, streptomycin and Fungizone. AX cells, derived from liposarcoma explants grown in mice, were maintained in F10 medium (Sigma) containing 10% fetal bovine serum (Sigma), penicillin, streptomycin and Fungizone [80]. To downregulate NumbL expression, short hairpins RNA (shRNA) against NumbL, Numb or scrambled sequence control in pB-RS vectors were obtained from Origene (Rockville, MD). We used TR311063 for the NumbL gene and TR311064 for the Numb gene. Both cell lines were transfected with shRNA plasmids and selected with 2 μg mL^−1^ of hygromycin. After selection, two of the four shRNA against NumbL were selected, shNbL2 (GGACAGCCAATAAAGGAAGAATATAATGG) and shNbL4 (AGATTTGTATTATACAAGGACAGCCAATA), while for Numb, we selected the following sequence: ATCATTCCGTGTCACAACAGCCACTGAAC after WB and qPCR analysis. NumbL was obtained by PCR and cloned in pBabe-puro using EcoRI and BamHI sites. A total of 10^6^ cells of HeLa and T47D cells were seeded in 10 cm^2^ plates, transfected with pBabe (EV or NumbL) 24 hours after seeding and selected with 1 μg mL^−1^ of puromycin.

For Western blotting detection, we used NumbL (Abcam, ab37500), Notch (Santa Cruz Biotechnologies, sc-6014-R, 1:200 dilution) and hnRNP C1/C2 (4F4) (Santa Cruz Biotechnologies, sc-32308, 1:400 dilution) antibodies. a-Tubulin (T9026, Sigma) was used as a loading control guide at 1:10000 dilution. Horseradish peroxidase-labeled rabbit anti-mouse (ab97046, Abcam, diluted 1:5000) and goat anti-rabbit (ab97051, Abcam, diluted 1:5000) secondary antibodies were used.

### Clonogenicity assays

For clonogenicity assays, 1000 cells were counted and seeded by triplicate in 10 cm^2^ plates for 12 days for HeLa and AX cells and 20 days for T47D cells. At least 50 colonies were counted by plate using light microscopy to distinguish between holoclones, meroclones and paraclones. For crystal violet staining, cells were fixed using PBS + 4% glutaraldehyde for 20′, washed twice with PBS and analyzed by microscope (Olympus CKX41 with integrated camera Olympus SC30, U-CMAD3) to detect holoclones, meroclones and paraclones. After that, the plates were dyed with a 1% crystal violet solution. Colony number was determined using ImageJ software. Both assays were performed in triplicate. To detect significant differences, Student's *t*-test was applied to each pair of samples, with a threshold of *p* < 0.05.

To obtain tumorspheres, cells were detached from the plate using a scraper and homogenized by pipetting. A total of 5000 cells of each line were counted and cultured by triplicate in Ultra-Low attachment multiwell plates (Corning) for 5 days in 1.5 mL complete MammoCult^TM^ medium (contains the MammoCult Basal medium, MammoCult Proliferation Supplement, fresh Hydrocortisone and Heparin; Stemcell technologies) at 37°C, 5% CO_2_. After that, cells were recovered and centrifuged, discarding the supernatant. Cells were trypsinized and counted again, being seeded again in Ultra-Low attachment conditions in a density of 5000 cells/well and cultured in 1.5 mL complete MammoCult media for other 5 days. Tumorspheres or aggregates were then visualized by microscope (Olympus CKX41 with an integrated camera Olympus SC30, U-CMAD3). To detect significant differences, this experiment was performed in triplicate, with Student's *t*-test applied to each pair of samples, with a threshold of *p* < 0.05.

HeLa and T47D cells were trypsinized and homogenized to obtain colonies using soft agar. A total of 5 × 10^4^ cells were suspended in 1.4% agarose D-1 Low EEO (Pronadisa, Alcobendas, Madrid, Spain) growth medium containing 10% FBS, disposed onto 1 mL of a solidified base of growth medium containing 2.8% agar prepared in 6-well plates. After 24 h, a medium containing 10% FBS was added to each 35-mm dish and replaced twice weekly. Colonies were scored after 15 days, and all values were measured in triplicate. Photographs were taken with a phase-contrast microscope (Olympus CKX41) with an integrated camera (Olympus SC30, U-CMAD3).

### Protein isolation and nuclei purification

HeLa and T47D cells were washed twice with PBS and lysed by sonication in RIPA lysis buffer (50 mM Tris-HCl, pH 7.5; 1% NP-40, 2 mM Na_3_VO_4_, 150 mM NaCl, 20 mM Na_4_P_2_O_7_, 100 mM NaF), supplemented with a complete protease inhibitor cocktail (P8340, Sigma) and phosphatase inhibitor cocktail (P5726, Sigma).

To detect nuclear NICD by WB, HeLa and T47D cells were trypsinized, washed twice with cold PBS, and incubated for 30′ in hypotonic buffer (20 mM HEPES-KOH, pH 7.5, 10 mM KCl, 1 mM Na-EDTA, 1 mM Na-EGTA, 1.5 mM MgCl_2_, 2 mM dithiothreitol (DTT)) supplemented with a complete protease inhibitor cocktail (P8340, Sigma) and a phosphatase inhibitor cocktail (P5726, Sigma). Afterward, cells were lysed by repeated passage through a 22G needle and centrifuged at 10000 g for 10′ at 4°C. The pellet was considered as a nuclei-enriched fraction and sonicated using complete RIPA buffer. This nuclei-enriched fraction was certified by WB due to the presence of hnRNP C1/C2 only in this fraction compared to the supernatant (cytoplasmic fraction, data not shown).

Total protein quantification was performed with Bio-Rad Protein Assay Dye Reagent concentrate.

### Analysis of gene transcription

Total RNA was purified using the ReliaPrep^TM^ RNA Tissue Miniprep System (Promega, Fitchburg, WI, USA) according to the manufacturer's instructions. Reverse transcription was performed with 3 μg of mRNA using the High-Capacity cDNA Reverse Transcription Kit (Life Technologies) according to the manufacturer's recommendations. To detect changes in gene expression, we used the following probes, all from Life Technologies: Hes1 (Hs00172878_m1), Hes5 (Hs01387463_g1), Hey1 (Hs01114113_m1), Klf7 (Hs00748636_s1), Id2 (Hs04187239_m1), Gli1 (Hs01110766_m1), Klf4 (Hs00358836_m1), Sox2 (Hs01053049_s1), Nanog (Hs04260366_g1), Oct4 (Hs00999632_g1), Bmi1 (Hs00995536_m1), NumbL (Hs00191080_m1), Numb (Hs01105433_m1) and Gapdh (Hs03929097_g1). The PCR reaction mixture (10 μL) contained 2 μL of 1/10 dilution reverse transcriptase reaction product, 5 μL TaqMan 2× Universal PCR Master Mix and 0.5 μL of the appropriate TaqMan Assay (20×) containing primers and a probe for the mRNA of interest. Polymerase chain reactions (PCR) were performed using the ABI Prism 7900HT sequence detection system (Applied Biosystems) to evaluate expression of the selected genes using the GoTaq^®^ Probe qPCR Master Mix, following the manufacturer's recommendations. The thermocycler parameters were 95°C for 10′ followed by 40 cycles of 95°C for 15′′ and 60°C for 1′. Relative changes in gene expression levels were calculated using the comparative threshold cycle (ΔΔCt) method. This method first subtracts the Ct (threshold cycle number) of the gene-average Ct of the housekeeping gene Gapdh to normalize the RNA amount. Finally, the ΔΔCt was calculated as the normalized average Ct of the test group vs the normalized average Ct of the Gapdh gene. This ΔΔCt value was raised to the power of 2 to calculate the degree of change. At least three independent experiments were conducted for each of the analyzed genes. The results are expressed as the percentage relative to EV, normalized as 100% for EV control. To detect significant differences, Student's *t*-test was applied to each pair of samples, with a threshold of *p* < 0.05.

### Fluorescence-activated cell sorting (FACS) analysis

HeLa and T47D cells were washed once with PBS and then harvested with 0.05% trypsin/0.025% EDTA. Detached cells were washed with PBS containing 2% FCS and 5 mM EDTA (wash buffer), resuspended in this buffer at 8000 cells μL-1 in a volume of 125 μL and then blocked for 10′ with 12.5 μL of FcR Blocking Reagent (MACS Miltenyi Biotec cat. #130-059-901). Combinations of fluorochrome-conjugated monoclonal antibodies obtained from MACS Miltenyi Biotec against human CD44 (APC; cat. #130-095-177), CD24 (PE: cat. #130-095-953) and CD133 (PE; cat. #130-098-826) or their respective isotype controls were added to the cell suspension at concentrations recommended by the manufacturer and incubated at 4°C in the dark for 30′. Labeled cells were washed in wash buffer, resuspended in 500 μL of wash buffer and analyzed on a FACS Canto II Analyzer cytometer.

### IC50 analysis

To determine whether NumbL knockdown confers an advantage to tumorigenicity, 5×10^3^ HeLa cells and 7.5×10^3^ T47D cells were seeded in 96-well plates and were treated 24 hours later. HeLa and T47D cells were treated with gemcitabine, doxorubicin, irinotecan, 5-flurouracil, vincristine and paclitaxel; T47D cells were specifically treated with capecitabine and sunitinib. Ninety-six hours later, cells were treated with MTS (MTS cell proliferation Assay Kit, Biovision, USA) for 2 hours, and the optical absorbance was measured at 490 nm.

To detect the stem cell population in T47D cells after treatment with vincristine, both T47D scr and shNbL2 were treated with a vincristine concentration of 1.5 nM, higher than the determined IC50 value for this drug. After 96 hours, cells were treated as previously described for analysis in the FACS Canto II Analyzer cytometer.

### Patients and clinical specimens

The present methylation study was performed in 47 patients following surgical resection for clinical early stage NSCLC. During the surgical procedure, the tumor and matched non-tumor tissue samples were collected from all patients and then immediately snap-frozen to −80°C for future use. The clinical features of patients with NSCLC are summarized in Supplementary Table S1. The NumbL methylation profile was also evaluated in lung tissue of a control cohort of 23 patients. The control cohort without lung cancer was composed of COPD (chronic obstructive pulmonary disease) patients and non-COPD subjects. A description of this cohort can be found on Supplementary Table S2. A written consent form was obtained from all participants. The study protocol and the use of human samples were approved by the Ethical Committee of the Virgen del Rocio University Hospital.

### DNA samples

Genomic DNA was extracted from tumor and matched non-tumor tissue samples by the QIAamp DNA mini kit (QIAGEN, Valencia, CA, USA). DNA was quantified using the QuantiFluor dsDNA system (Promega, Madison, WI, USA) according to the manufacturers' instructions.

### Illumina 450 K methylation

The Illumina Infinium Human Methylation 450 BeadChip (Illumina Inc., San Diego, CA) was used to interrogate 485,000 methylation sites across the genome per sample at single-nucleotide resolution. It covers 96% of the CpG islands, with additional coverage in island shores and the flanking regions. We treated 500 ng of DNA with sodium bisulfate using the EZ DNA Methylation™ Kit and cleaned the DNA with the ZR-96 DNA Clean-up Kit™ (EZ DNA, Zymo Research, Irvine, CA) before standard Illumina amplification, hybridization, and imaging steps. The resulting intensity files were analyzed with Illumina's GenomeStudio, which generated β-scores (i.e., the proportion of total signal from the methylation-specific probe or color channel).

### Methylome data processing

Methylome data were processed using the RnBeads R package [81]. After a quality check, the probe median intensity was normalized with the SWAN method [82] and converted to beta values. The probes were tested for differential methylation with the limma method, a linear model followed by empirical Bayes methods for the comparisons of interest [83]. The CpG status (hypo- versus hyper-methylated) and CpG chromosomal location were realized using the Circos data visualization software [84]. DNA methylation data were visualized by the Wash U Epigenome Browser [85].

## SUPPLEMENTARY MATERIALS FIGURES AND TABLES


