# Supplementary material for: Numb-like (NumbL) downregulation increases tumorigenicity, cancer stem cell-like properties and resistance to chemotherapy
Source: Oncotarget. 2016 Aug 23;7(39):63611–28. doi: 10.18632/oncotarget.11553 (PMC5325389; doi:10.18632/oncotarget.11553)
Supplement: Supplementary file 1 [file oncotarget-07-63611-s001.pdf]

Numb-like (NumbL) downregulation increases tumorigenicity, cancer stem cell-like properties and resistance to chemotherapy

Supplementary Materials

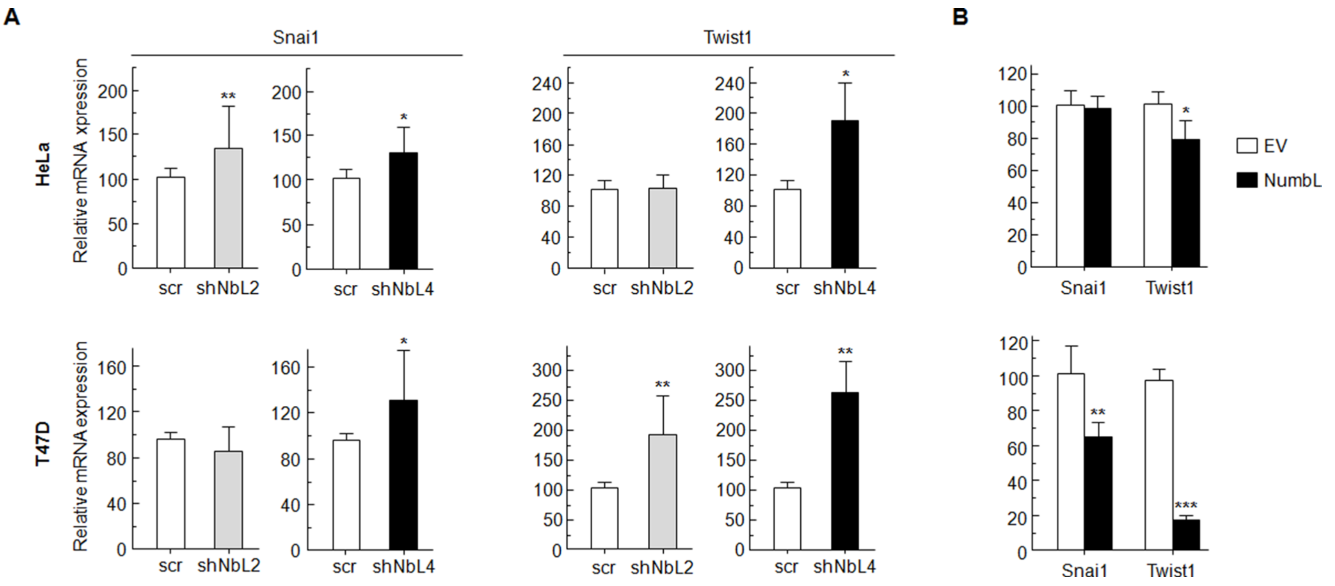

Supplementary Figure S1: (**A**) NumbL downregulation activates EMT signaling related genes. (**B**) NumbL overexpression inhibits EMT signaling related genes. RT-qPCR was used to measure Snai1 and Twist1 genes according to M&M. All experiments were repeated a minimum of three independent times in triplicate. All figures include Student's *T* test for statistical analysis of the data. * = $p < 0.05$; ** = $p < 0.01$; *** = $p < 0.001$.

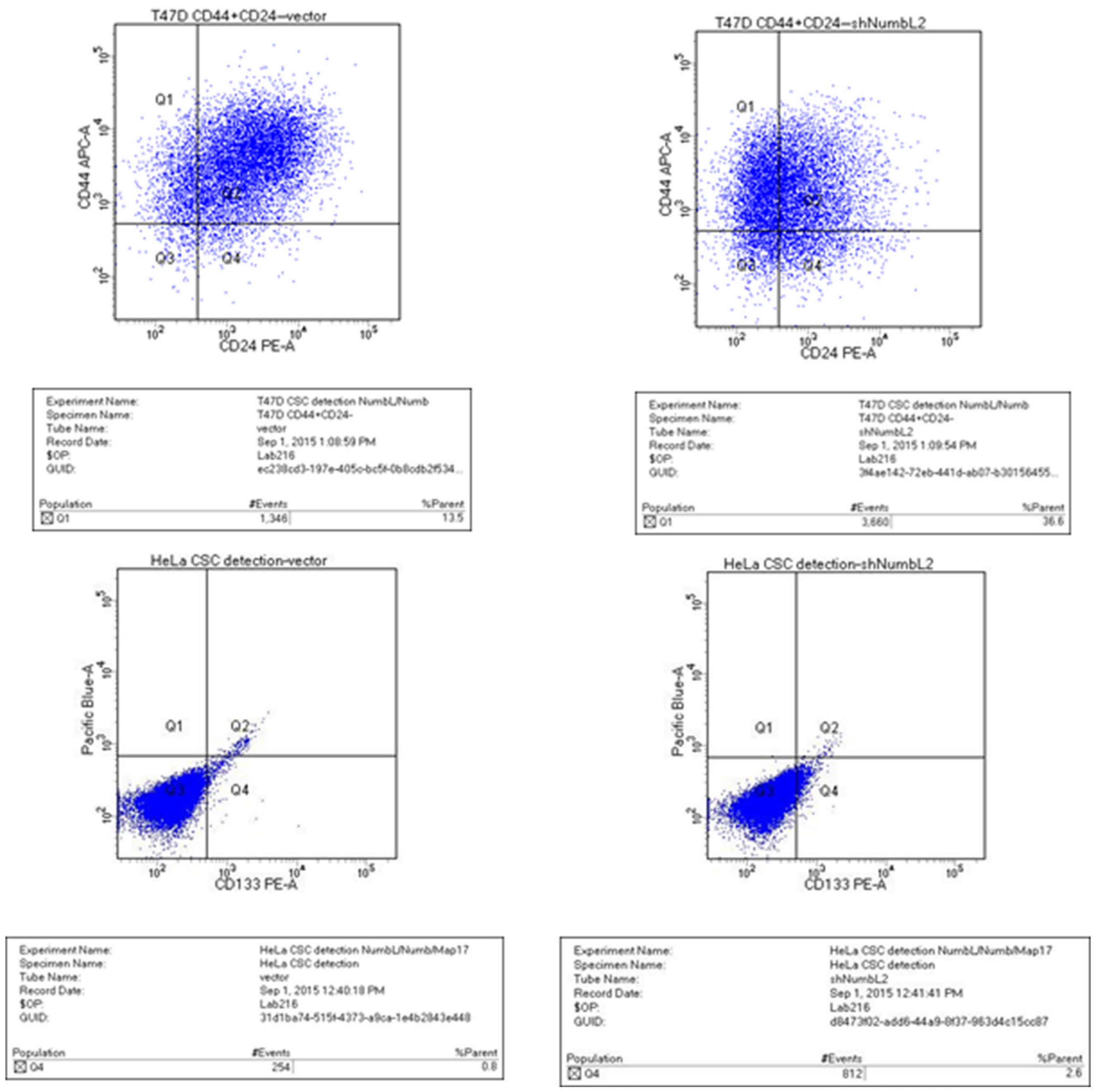

Supplementary Figure S2: The FACs plots of CD44$^+$/CD24$^-$ and CD133$^+$ FACS of T47D and HeLa cells, respectively. Data are shown in Figure 3B.

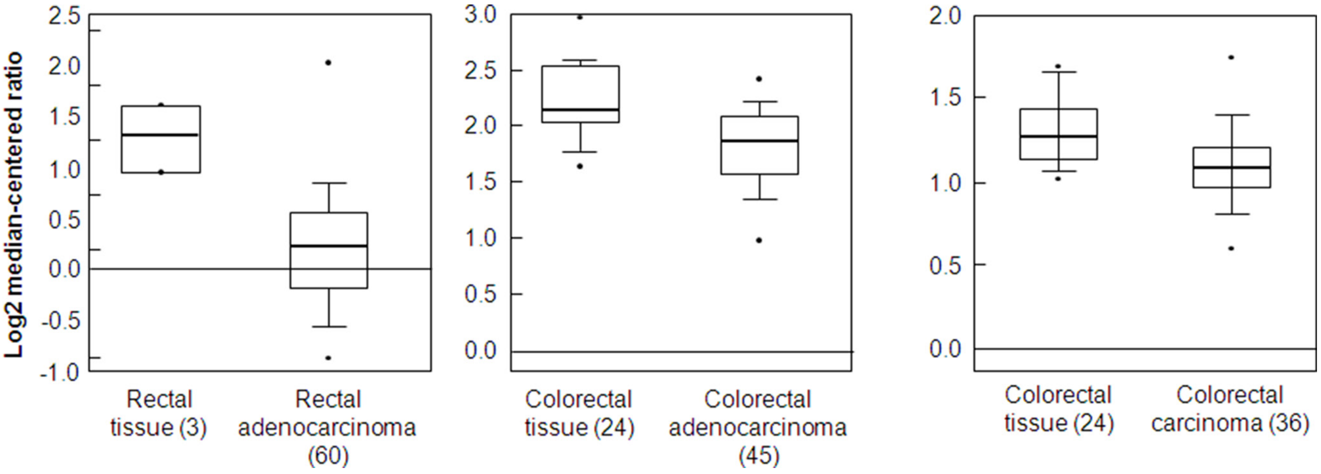

Supplementary Figure S3: Relative levels of the NumbL transcript in three different databases of colon cancer. Data obtained from Oncomine.

Supplementary Table S1: Characteristics of the study cohort for Table 2: Methylation of the NumbL promoter in lung cancer

	Patients (*N* = 47)
**Gender**	
Male	76.6 (36)
Female	23.4 (11)
**Age (years)**	67 [60-73]
**Smoking status**	
Smokers	40.4 (19)
Ex-smokers	44.7 (21)
Non-smokers	14.9 (7)
**Pack years**	41.0 [20.0-65.7]
**Histology**	
Lung adenocarcinoma	57.4 (27)
Squamous cell carcinoma	42.6 (20)
**Staging**	
Stage I	40.5 (19)
Stage II	38.3 (18)
Stage III-IV	21.2 (10)
**Subjects with COPD**	42.6 (20)
	**Controls (*N* = 23)**
**Gender**	
Male	87.0 (20)
Female	13.0 (3)
**Age (years)**	35 [21-62]
**Smoking status**	
Smokers	30.4 (7)
Ex-smokers	21.7 (5)
Non-smokers	47.8 (11)
**Pack years**	2.0 [2.0-20.0]
**Subjects with COPD**	17.4 (4)

Continuous variables are expressed as the median [interquartile range (IQR)] and categorical variables are expressed as the number of cases (%).

Supplementary Table S2: Data from figure 8 analysis

Figure 6 panel	Tumor	Endpoint	Hazard ratio [95% CI]	Dataset
A	Colorectal	Disease Free survival (DFS)	0.20 [0.03–1.30]	GSE17537
B	Colorectal	Overall survival (OS)	0.26 [0.04–1.87]	GSE17537
C	Colorectal	Disease specific survival (DSS)	0.21 [0.04–1.01]	GSE17537
D	Breast	Relapse Free survival (RFS)	0.76 [0.36–1.59]	GSE1456-GPL97
E	Breast	Overall survival (OS)	0.88 [0.47–1.66]	GSE1456-GPL97
F	Breast	Disease specific survival (DSS)	0.62 [0.33–1-19]	GSE1456-GPL97
G	Lung Adc	Disease Free survival (DFS)	0.19 [0.06–0.61]	GSE31210
H	Lung Adc	Overall survival (OS)	0.19 [0.08–0.47]	GSE31210

Include public dataset used.
